# Intraspecific Variation and Phylogenetic Relationships Are Revealed by ITS1 Secondary Structure Analysis and Single-Nucleotide Polymorphism in *Ganoderma lucidum*

**DOI:** 10.1371/journal.pone.0169042

**Published:** 2017-01-05

**Authors:** Xiuqing Zhang, Zhangyang Xu, Haisheng Pei, Zhou Chen, Xiaoyan Tan, Jing Hu, Bin Yang, Junshe Sun

**Affiliations:** 1 Beijing Advanced Innovation Center for Food Nutrition and Human Health, College of Food Science and Nutritional Engineering, China Agricultural University, Beijing, China; 2 Chinese Academy of Agricultural Engineering, Beijing, China; 3 Department of Biological Systems Engineering, Washington State University, Richland, WA, United States of America; Jilin University, CHINA

## Abstract

*Ganoderma lucidum* is a typical polypore fungus used for traditional Chinese medical purposes. The taxonomic delimitation of *Ganoderma lucidum* is still debated. In this study, we sequenced seven internal transcribed spacer (ITS) sequences of *Ganoderma lucidum* strains and annotated the ITS1 and ITS2 regions. Phylogenetic analysis of ITS1 differentiated the strains into three geographic groups. Groups 1–3 were originated from Europe, tropical Asia, and eastern Asia, respectively. While ITS2 could only differentiate the strains into two groups in which Group 2 originated from tropical Asia gathered with Groups 1 and 3 originated from Europe and eastern Asia. By determining the secondary structures of the ITS1 sequences, these three groups exhibited similar structures with a conserved central core and differed helices. While compared to Group 2, Groups 1 and 3 of ITS2 sequences shared similar structures with the difference in helix 4. Large-scale evaluation of ITS1 and ITS2 both exhibited that the majority of subgroups in the same group shared the similar structures. Further Weblogo analysis of ITS1 sequences revealed two main variable regions located in helix 2 in which C/T or A/G substitutions frequently occurred and ITS1 exhibited more nucleotide variances compared to ITS2. ITS1 multi-alignment of seven spawn strains and culture tests indicated that a single-nucleotide polymorphism (SNP) site at position 180 correlated with strain antagonism. The HZ, TK and 203 fusion strains of *Ganoderma lucidum* had a T at position 180, whereas other strains exhibiting antagonism, including DB, RB, JQ, and YS, had a C. Taken together, compared to ITS2 region, ITS1 region could differentiated *Ganoderma lucidum* into three geographic originations based on phylogenetic analysis and secondary structure prediction. Besides, a SNP in ITS 1 could delineate *Ganoderma lucidum* strains at the intraspecific level. These findings will be implemented to improve species quality control in the *Ganoderma* industry.

## Introduction

*Ganoderma lucidum* (Chizhi) is a wood-rotting fungus that is also a prestigious medicine [1] and has been continuously used in Asia for approximately two thousand years [2]. The active ingredients of *Ganoderma lucidum*, extracted using hot water or ethanol, include polysaccharides [3–5], proteins [6,7] and ganoderic acid [8,9]. These ingredients can be effective in the treatment of cancer, hypertension and viral infections, since numerous reports have indicated that *Ganoderma lucidum* has anti-tumor [10–12], anti-aging [13,14], immune system-enhancing, [15,16] and anti-hypertension [16–18] activities. Therefore, it has been widely planted and used in human health products, such as dietary supplements. The worldwide trade value of *Ganoderma lucidum* and its derived products is approximately 2.5 billion US dollars per year [2]. Due to the high demand of medical use and the potential profits, the identification and quality control of *Ganoderma lucidum* is a critical point for further industry development.

A debate appears to persist in the delineation of *Ganoderma* species. Karsten [19] from the UK first established the *Ganoderma* genus in 1881. After that, morphologically similar fungi were identified in Asia and Europe and given the same name of *Ganoderma lucidum* [20]. Due to geographical isolation, there are several morphological discrepancies between the Asian and European species, such as color, thickness and context in the fruit body [6]. Thus, Chinese scholars considered the *Ganoderma lucidum* species of Asia to differ from those of Europe. The typical *Ganoderma* in China is proposed as *Ganoderma lingzhi* [6,21] by taxonomists, whereas the traditional *Ganoderma* species were named as *Ganoderma lucidum* (Chi-Zhi) in the Chinese Pharmacopoeia. However, the *Ganoderma* species delineation is still disputed.

Traditional identification of *Ganoderma lucidum* is based on fruit body identification and culture description, which collectively are time-consuming. In the attempt to overcome the difficulties of basidiocarp morphology in species identification, alternative methods have been investigated [22], including culture characteristics [20,23,24], isozyme profiles [25] and DNA-based techniques [26–28]. While antagonism is stably used in fungus breeding [29] and in fungal pathogen defense [30], which occurs when fungal hyphae with distinct genetic backgrounds come into contact with each other.

DNA molecular technologies are widely used and considered as the most effective tools in the delineation of fungi, given their genetic stability and variance among species. Different molecular technologies have been published for the purpose of the delineation of *Ganoderma*, including internal transcribed spacer (ITS) [31], simple sequence repeat (SSR) [32], random amplified polymorphic DNA (RAPD) [33], amplified fragment length polymorphism (AFLP) [34], and mitochondrial DNA (mtDNA) [27] sequencing. Among these technologies, ITS sequencing of the nuclear rDNA is the most frequently used in *Ganoderma* identification. The entire ITS sequence consists of two variant spacers (ITS1 and ITS2) and the conserved 5.8s gene. Given the conserved sequences, the ITS region [35] and ITS2 region [36] have been recognized as DNA barcode sequences for species identification. The ITS2 region was identified as the barcode for the delineation of *Ganoderma lucidum* by Liao et al. [37]. However, regarding ITS1, which is also considered as a barcode candidate for fungi [38]. In *Ganoderma*, intra-strain heterogeneity in the ITS sequences of the *Ganoderma applanatum var*. *gibbosum*, *Ganoderma fornicatum*, *Ganoderma japonicum* and *Ganoderma neojaponicum* strains was previously reported [39]. Kinge et al. [22] reported that heterogeneity was mostly observed in the ITS1 region, and Wang et al. [40] concluded that ITS1 represents a better DNA barcode than ITS2 for eukaryotic species based on a systematic comparison. These studies indicated that ITS1 could be another barcoding marker candidate in fungi delineations.

Here, we reported that the ITS1 region could differentiate *Ganoderma lucidum* into three geography-originated groups that are associated with secondary-structure variance. And we also compared the difference between ITS1 and ITS2 regions with phylogenetic analysis and large-scale secondary structures prediction. In a specific analysis with Chinese spawn strains of *Ganoderma lucidum*, we found that strain compatibility is correlated with the single-nucleotide polymorphism (SNP) site in ITS1. These findings indicate that ITS1 could be a barcode marker candidate and thus support a novel method for *Ganoderma lucidum* delineation.

## Materials and Methods

### Organism and inoculation

Seven *Ganoderma lucidum* strains (including 203, HZ, RB, TK, YS, JQ and DB) were obtained from the Keda Company in Zhejiang province. These strains were used as spawns for commercial *Ganoderma lucidum* fruit bodies. The cultures were maintained with potato dextrose agar (PDA, 1 L, 200 g potatoes, 2% glucose, 15 g agar) slopes. The slopes were inoculated and incubated at 28°C for 15 days and stored at 4°C. To prepare the inoculum, the mycelium of *Ganoderma lucidum* was transferred to a petri dish containing PDA medium at 28°C for 15 days. Mycelium agar discs (1 cm) were obtained with a self-designed cutter and were used as inoculum in 100-mL shake flasks that contained 50 mL of PDA liquid medium. The flasks were incubated on a rotary shaker at 150 rev/min at 28°C for 15 days.

### DNA extraction and amplification

The mycelia were harvested by vacuum filtration. The *Ganoderma lucidum* mycelia were ground with liquid nitrogen, and the genomic DNA was then extracted following the CTAB method [34]. The ribosomal DNA ITS (rDNA-ITS) region was amplified with the universal primers ITS-1F (5’-CTTGGTCATTTTAGAGGAAGTAA-3’) and ITS-4B (5’-CAGGAGACTTGTACACGGTCCA-3’) [41] using Taq PCR MasterMix (TIANGEN, CO., LTD.). The PCR parameters were as follows: 94°C for 5 min; 30 cycles of 94°C for 30 s, 55°C for 30 s and 72°C for 1 min, and a final extension step of 10 min at 72°C. Electrophoretic analysis of the PCR products revealed the presence of a single DNA band of approximately 0.7 kb, which was purified and subjected to Sanger sequencing (BGI Tech Corporation, http://www.genomics.cn/).

### Strain antagonism

Mycelium agar discs (1 cm) were obtained from fresh PDA plates and transferred to accelerating medium plates (1 L, sucrose 35 g, peptone 5 g, yeast extract 2.5 g, KH_2_PO_4_·H_2_O 1 g, MgSO_4_·7H_2_O 0.5 g, VB_1_ 0.05 g, agar 15 g) [9]. Each plate contained three agar discs of different *Ganoderma lucidum* strains and was incubated at 28°C for 7 days. Antagonistic streaks were photographed using a digital single lens reflex camera (Canon, 70D).

### Sequence annotation and alignment

The sequenced *Ganoderma lucidum* ITS sequences derived from this study (GenBank accession nos: KX589244-KX589250) were annotated based on Profile Hidden Markov Models (HMMs) [42]. The ITS1 and ITS2 regions from the ITS sequences were annotated and extracted using the HMMER suite [43] (version 3.1b) and the ITSx package [44] (version 1.0.11). The ITS1 annotated sequences were then reevaluated by NCBI BLAST (https://blast.ncbi.nlm.nih.gov/Blast.cgi). The ITS2 annotated sequences were then reevaluated by ITS2 database (http://its2.bioapps.biozentrum.uni-wuerzburg.de/) [45]. A set of 378 unique *Ganoderma lucidum* rDNA-ITS sequences was retrieved from the GenBank NT (Nucleotide) database, including 52 individual ITS1 sequences and 50 individual ITS2 sequences. All of the downloaded sequences were annotated following the above procedure. The sequenced and annotated ITS1 and ITS2 sequences of *Ganoderma lucidum* were combined separately for further analyses. Multiple sequence alignments of annotated ITS1 sequences of seven spawn *Ganoderma lucidum* strains were performed using DNAMAN software (version 6.0; Lynnon BioSoft, Canada).

### Phylogenetic analyses

All ITS1 sequences of *Ganoderma lucidum* were aligned using the MUSCLE method [46]. The best fitting substitution model was estimated based on the Akaike information criterion (AIC) using the built-in model selection option of MEGA 6.0 [47]. The Kimura 2-parameter (K2P) model was selected for analysis of the ITS1 region. A maximum likelihood (ML) tree was constructed using MEGA 6.0 with the following parameters: the bootstrap method was conducted with 1000 replicates, the substitution model was Kimura 2-parameter (K2P), the rates among sites were uniform, and gaps were treated as missing data (complete deletion). Other parameters followed the default settings. *Trametes versicolor* EF524049.1 [48] and *Trametes versicolor* HM008935.1 [49] were set as outgroups for both ITS1 and ITS2 phylogenetic analysis. All ITS2 sequences of *Ganoderma lucidum* were also aligned by the MUSCLE method [45]. The Kimura 2-parameter (K2P) model was selected based on the Akaike information criterion (AIC) for analysis of the ITS2 region. A maximum likelihood (ML) tree of ITS2 sequences was constructed using MEGA 6.0 with the same parameters of ITS1 sequences. The trees of ITS1 and ITS2 sequences were both visualized using FigTree [50] (version 1.4.2) and the Interactive Tree Of Life (iTOL) web server (http://itol.embl.de/) [51].

### Weblogo analyses

The Weblogo program [52] was used to generate sequence logos for assessing sequence conservation within ITS1 and ITS2 and the relative frequencies of the nucleotides at each position. Combined ITS1 and ITS2 sequences of *Ganoderma lucidum* were aligned on MUSCLE [45] version 3.8 using the EMBL-EBI [53] web servers (http://www.ebi.ac.uk/Tools/msa/muscle/). Sequence alignments were uploaded and analyzed on Weblogo web servers (http://weblogo.berkeley.edu/) following the default parameters. Outgroups were removed from the alignment prior to generating the sequence logos.

### Secondary structure analyses

Annotated ITS1 sequences were folded by energy minimization using the RNAfold web server (http://rna.tbi.univie.ac.at/) [54] and the helix-wise divide and conquer approach proposed by Koetschan et al. [55]. Briefly, the latter method divides a sequence into several parts according to the presumed locations of the helices. Each part was folded separately and was integrated afterward to build the full structure. The sequences used as ITS1 secondary structure analysis for Group 1, Group 2 and Group3 were EU498090.1, EU021461.1, and FJ687271.1, respectively. Annotated ITS2 sequences were folded by energy minimization using the ITS2 database web server (http://its2.bioapps.biozentrum.uni-wuerzburg.de/). The sequences used as ITS2 secondary structure analysis for Group 1, Group 2 and Group3 were EU498090.1, EU021461.1, and FJ379263.1, respectively. Putative secondary structures were finally visualized using PseudoViewer 3 [56] and Adobe Illustrator® v. 16.0.1.

## Results

### Sequence annotation

To analyze the phylogenetic relationships of *Ganoderma lucidum* species, a total of 241 ITS1 sequences, including 234 downloaded from NCBI database and 7 sequenced ITS whole sequences, were successfully annotated in the HMMER suite. Besides, a total of 153 ITS2 sequences, including 146 downloaded from NCBI database and 7 sequenced ITS whole sequences, were successfully annotated based on the HMMER suite and ITS2 database.

### Phylogenetic analysis of ITS1 and ITS2 sequences

**Fig 1:**
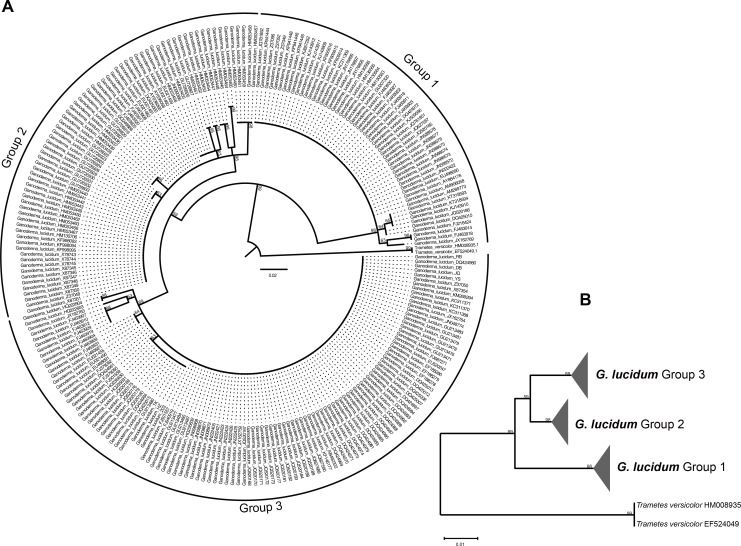
Molecular phylogeny of the *Ganoderma lucidum* species used in this study based on the ITS1 rRNA region. (A) The 50% majority rule consensus tree (Cladogram) from maximum likelihood analyses based on 241 sequences of the ITS1 rRNA region. (B) Three groups (1–3) are identified among *Ganoderma lucidum* sequences. Branch support is noted on branches (only for the collapsed branches). Both trees are rooted to *Trametes versicolor*.

Generally, the species of *Ganoderma lucidum* could be separated into three groups by phylogenetic analysis of ITS1 sequences (Fig 1). Seven *Ganoderma lucidum* strains were differentiated in the same group (Group 3). However, *Ganoderma lucidum* 203, HZ and TK (KX589244, KX589246, KX589249, S1 Table) were separated into a subgroup, which indicated their homology, and *Ganoderma lucidum* DB, JQ, RB, and YS (KX589245, KX589247, KX589248, KX589250, S1 Table) formed another subgroup.

Group 1 is composed of 56 strains originally determined as *Ganoderma lucidum*, including 12 strains from Italy, 8 strains from India, 6 strains from China, 5 strains from Russia, 4 strains from Armenia, 3 strains from France, 2 strains from the UK, and 1 strain from Canada, Poland, Sweden, Slovenia, Czech Republic, the USA, Norway and Finland, respectively. This clade received 98% support in the bootstrap analysis. Two Chinese strains, FJ216424 and DQ425010, served as a sister group of Group 1, and they were grouped together with a bootstrap of 64%. The strains JQ520185, KJ143910, KT318594 and KT318593, which originated from Canada, China and two unknown regions, served as a sister group with 65% bootstrap support. Strain JX162769 served as a sister group with two Indian strains, FJ463918 and FJ463914, with a bootstrap of 63%. In addition, the latter two strains served as a sister group with the strong bootstrap support of 98%.

The Group 2 cluster of *Ganoderma lucidum* originated from India, Taiwan, the Philippines and mainland China, with 98% support in the bootstrap analysis. Group 2 is composed of 73 strains, with 54 strains originating from India, 11 strains originating from Taiwan, 3 strains originating from mainland China and 1 strain originating from the Philippines. Indian strains formed 8 sister groups with bootstrap support ranging from 63% to 98%.

Group 3 contained 112 strains of *Ganoderma lucidum* from mainland China, Japan, Korea, India, and Bangladesh, with 89% support in the bootstrap analysis. *Ganoderma lucidum* commercial spawns 203, HZ, TK, (KX589244, KX589246, KX589249) and 12 strains that originated from mainland China and Korea, respectively, served as sister groups with 63% bootstrap support. Indian strains FJ463904, FJ463909, FJ463921 and FJ463931 served as sister groups with 64% bootstrap support. In addition, the JX162763 strain originated from mainland China and separated with five strains that originated from India with 63% bootstrap support. In addition, two *Ganoderma lucidum* strains with unknown origins formed a sister group with strong bootstrap support (98%). Seven spawn *Ganoderma lucidum* strains were in the same group. However, these strains also could be separated into two different subgroups: one including strains 203, HZ and TK, (KX589244, KX589246, KX589249) and the other including strains DB, JQ, RB, and YS (KX589245, KX589247, KX589248, KX589250).

**Fig 2:**
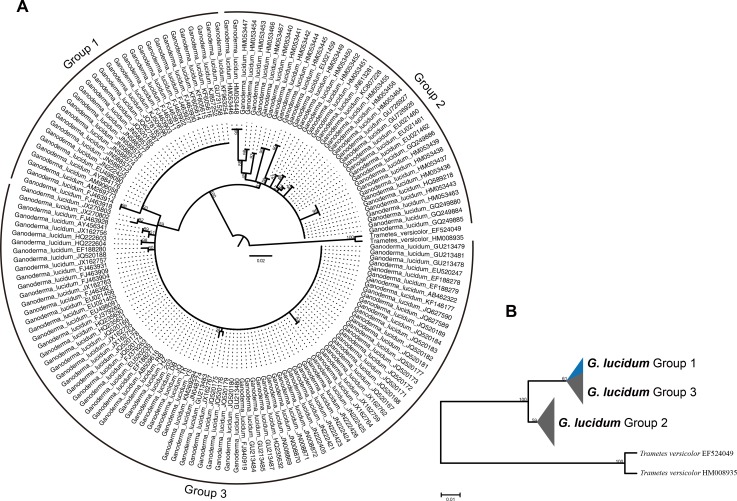
Molecular phylogeny of the *Ganoderma lucidum* species used in this study based on the ITS2 rRNA region. (A) The 50% majority rule consensus tree (Cladogram) from maximum likelihood analyses based on 153 sequences of the ITS2 region. (B) Two groups (1,3 and 2) are identified among *Ganoderma lucidum* sequences. Group 1 (blue part) and Group 3 as the subgroup formed the same group. Branch support is noted on branches (only for the collapsed branches). Both trees are rooted to *Trametes versicolor*.

Regarding ITS2 sequences, the species of *Ganoderma lucidum* could only be separated into two groups by phylogenetic analysis (Fig 2). Seven *Ganoderma lucidum* spawn strains were differentiated in the same group (Group 3).

Group 1 and Group 3 together formed one group (Fig 2A and 2B). Regarding Group 1, it is composed of 30 *Ganoderma lucidum* strains, including 7 strains from Italy, 8 strains from India, 2 strains from Armenia, 4 strains from France, and 1 strain from China, UK, Canada, Poland, and Sweden, respectively. This clade received 93% support in the bootstrap analysis. Two Chinese strains, FJ463914 and FJ463918, served as a sister group of Group 1, and they were grouped together with a bootstrap of 96%.

Group 3 contained 84 strains of *Ganoderma lucidum* from mainland China, Japan, Korea, India, and Bangladesh, with 59% support in the bootstrap analysis. *Ganoderma lucidum* commercial spawns 203, HZ, TK, RB, DB, YS, and JQ were served as the same group with other 71 strains. (KX589244, KX589246, KX589249, KX589248, KX589245, KX589250, KX589247). Indian strain FJ463928 and an unknown originated strain AY456341 served as sister groups with 89% bootstrap support. EF188280 originated from China and JQ520188 originated from Thailand served as sister groups with 60% bootstrap support. Also, the JQ520175 strain originated from Korea and JX162767 with unknown origination served as a sister group with 60% bootstrap support. In addition, JX162759 and JX162762 originated from mainland China served as a sister group with 87% bootstrap support.

The Group 2 cluster of *Ganoderma lucidum* originated from India and Taiwan with 61% support in the bootstrap analysis. Group 2 is composed of 38 strains, with 33 strains originated from India and 3 strains originated from Taiwan. Indian strains formed 9 sister groups with bootstrap support ranging from 86% to 99%.

### Characterizing the DNA sequences of ITS1 and ITS2 DNA sequence logos

**Fig 3:**
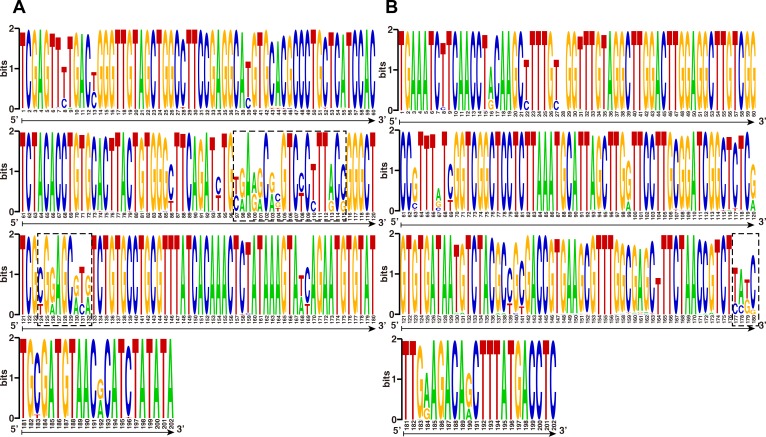
**DNA Weblogo of the (A) ITS1 and (B) ITS2 regions in strains of *Ganoderma lucidum*.** Regions represent potential nucleotide variance. Major variance regions are enclosed in boxes.

**Fig 4:**
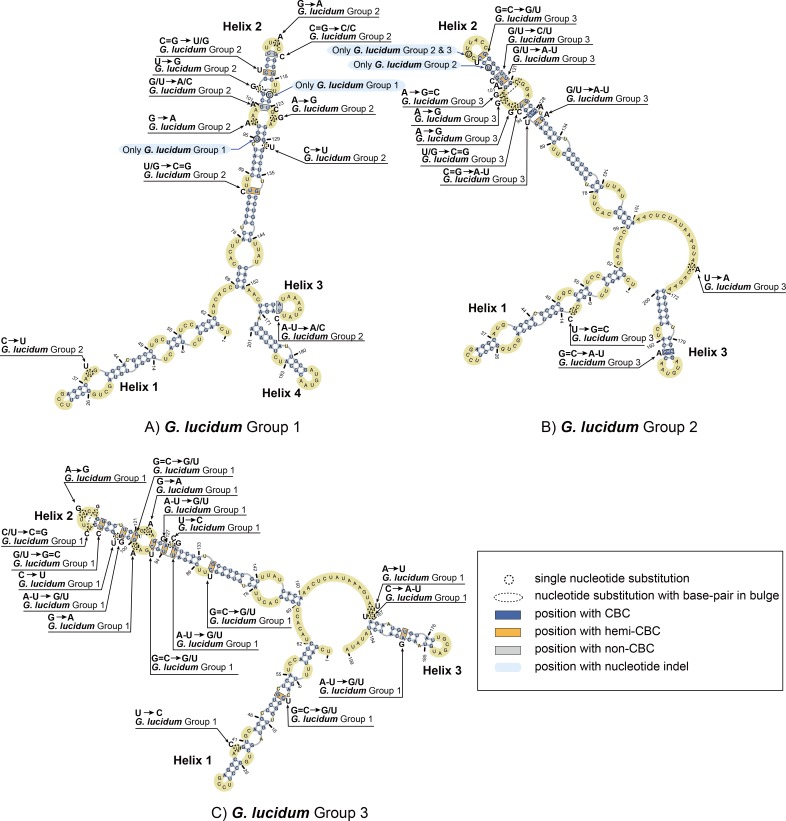
Predicted secondary structure models of the ITS1 rRNA molecule of *Ganoderma lucidum*. Three helices commonly found in the 2D structure of Groups 2 and 3 are numbered from Helix 1 to Helix 3. Four helices found in the 2D structure of Group 1 are numbered from Helix 1 to Helix 4. All substitutions recorded among three groups of *Ganoderma lucidum* are mapped on the 2D models.

DNA sequence logos were constructed for the ITS1 segments to visualize conserved regions and sequence variance (Fig 3A). There were two GC-rich regions within helix 1 (positions 3–62) and helix 2 (positions 70–144). These regions were potential hotspots for compensatory changes, including nucleotide substitutions and indels. Notably, the indels were responsible for the expansion or contraction of the helix structure (Fig 4). There were two main variable regions, both located within helix 2, including nucleotide positions 97 to 115 and 124 to 132. The nucleotide substitutions mainly consisted of the 13th C to T and the 9th A to G changes. The C to T substitutions were scattered within the ITS1 region, whereas A to G substitutions mainly occurred in the variable region of helix 2. There are helical-loop regions near the ITS1 starts and termini in which the nucleotides are highly conserved.

**Fig 5:**
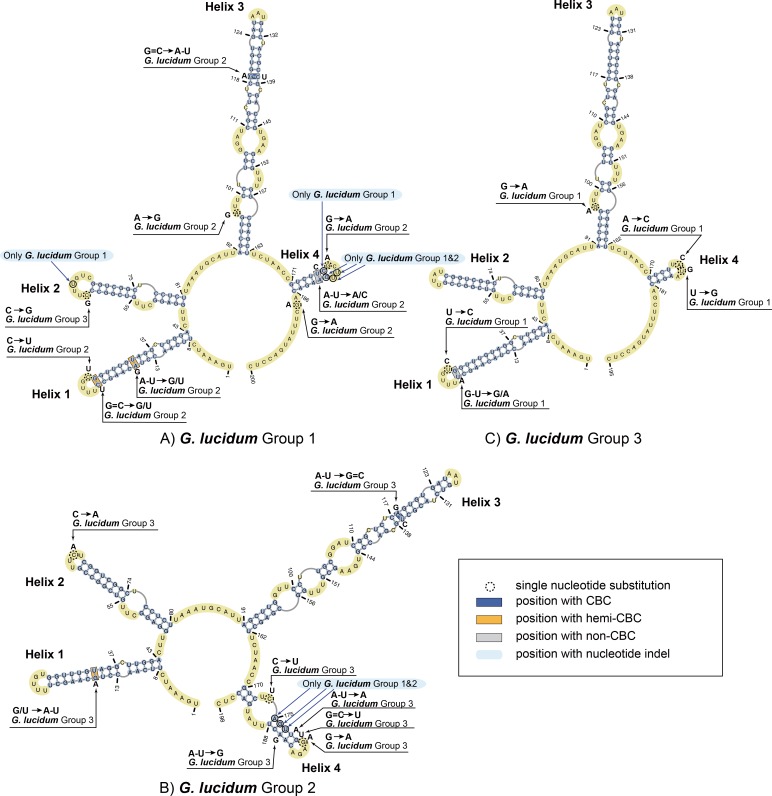
Predicted secondary structure models of the ITS2 rRNA molecule of *Ganoderma lucidum*. Four helices commonly found in the 2D structure of Groups 1, 2 and 3 are numbered from Helix 1 to Helix 4. All substitutions recorded among three groups of *Ganoderma lucidum* are mapped on the 2D models.

DNA sequence logos were constructed for the ITS2 segments to visualize conserved regions and sequence variance (Fig 3B). In general, seldom compensatory changes of ITS2 segments were found compared to ITS1 segments. There were three GC-rich regions within helix 1 (positions 26–43), helix 2 (positions 48–75), and helix 3 (positions 94–163). However, these regions were less potential hotspots for compensatory changes compared to corresponding ITS1 segments (indicating in Fig 5). Notably, the major potential hotspots for compensatory changes were mainly located in helix 4, which formed the main variable region from positions 177–180. The C to T and G to A substitutions were scattered within the ITS2 region. The nucleotides are highly conserved in helical-loop regions near the ITS2 starts and termini.

### Prediction and description of a common core in secondary structure

ITS1 sequences of three groups of *Ganoderma lucidum* were folded in the RNAfold program following the energy minimization method. Three representative structures are presented in Fig 4. For Group 1, 47 ITS1 sequences shared the same secondary structures which located in the main branch of Group 1 started from AM268773.1 to JQ781852.1 (Fig 1). Also for Group 2, 46 ITS1 sequences shared the same secondary structures started from EU021459.1 to GU726921.1 and GU726928.1 to X87351.1 in the main branch of Group 2 (Fig 1). In addition, for Group 3, 85 ITS1 sequences shared the same secondary structures from EU021455.1 to DQ424991.1 and FJ501557.1 to RB located in the main branch of Group 3 (Fig 1). All these sequences in each group shared the same secondary structures. Therefore, a sequence from these sharing parts of each group was chosen as the representative to compare secondary structures and the nucleotide variances of three groups were analyzed based on multi-alignments. The sequences used as representative secondary structure analysis for Group 1, Group 2, and Group3 were EU498090.1, EU021461.1, and FJ687271.1, respectively.

For ITS1 secondary structures, all three structures consisted of a central core formed by three (Groups 2 and 3) or four (Group 1) helices. The central core of Group 1 consists of two short helices (nt 156 to 171 and 172 to 201) near the 3’ terminus and two long helices (nt 3 to 62 and 69 to 152) near the 5’ terminus and the middle of the whole secondary structure. The central core of Group 2 consisted of a short helix (nt 172 to 200) near the 3’ terminus and two long helices (nt 3 to 62 and 69 to 151) near the 5’ terminus and the middle of the whole secondary structure. The central core of Group 3 consists of a short helix (nt 167 to 194) near the 3’ terminus and two long helices (nt 3 to 62 and 69 to 150) near the 5’ terminus and the middle of the whole secondary structure.

Specifically, helix 1 of three *Ganoderma lucidum* groups is approximately 60 nt long and consists of a long hairpin structure with four (Groups 1 and 2) or five (Group 3) bulges and a small loop. The primary sequence of helix 1 was conserved in length. Furthermore, the 2D structure was also conserved, with only one or two variable nucleotides appearing in bulges (indicated in Fig 4) among the three groups of *Ganoderma lucidum*. Notably, one hemi compensatory base change (hemi-CBC) site appeared in helix 1 of Group 3.

Helix 2 was the longest among all of the helices. This helix consisted of a long hairpin structure with five (Groups 1 and 3) or six (Group 2) bulges and a small loop. The primary sequences of helix 2 in the three groups were 84, 83 and 82 nt in length, respectively. Variations of the 2D structure of helix 2 were observed, with 9- to 14-nt polymorphisms among the three groups. Specific 2-, 2- and 1-nt indels could be observed in the alignment of ITS1 sequences of Groups 1, 2 and 3, respectively. Helix 2 contains 2 to 7 hemi compensatory base changes (hemi-CBCs) and one compensatory base change (CBC) and non-compensatory base change (non-CBC). The major patterns of nucleotide variances were C to U and G to A and *vice versa*. Nucleotide variances seldom exhibited different patterns, such as U to G, G to C or U to C.

Helix 3 of Group 1 was a short helix approximately 16 nt in length, including a short base-pair region and an 8-nt loop. A non-CBC appeared in the joint of base-pair and loop region. Helix 3 of Groups 2 and 3 and helix 4 of Group 1 varied in sequence length from 28 (Group 3) to 29 nt (Groups 1 and 2). Bulge variances were also discovered (1 in Groups 1 and 2 and 2 in Group 3). Two nucleotide variances in Groups 2 and 3 included a CBC and a hemi-CBC.

**Fig 6:**
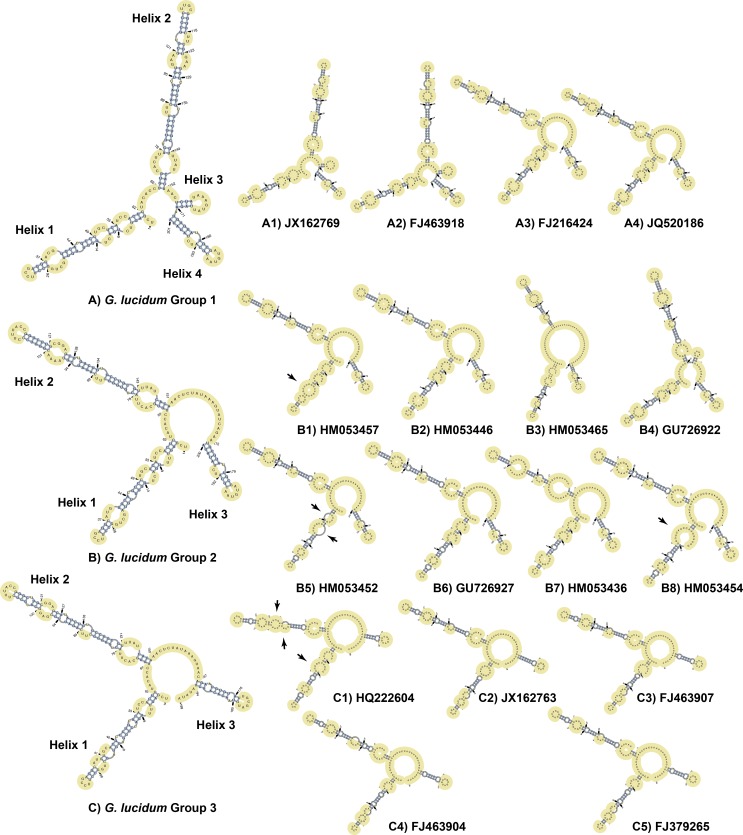
Large-scale evaluation of the ITS1 rRNA secondary structure models. A) *Ganoderma lucidum* Group 1 with four representative secondary structures of each subgroup. B) *Ganoderma lucidum* Group 2 with eight representative secondary structures of each subgroup. C) *Ganoderma lucidum* Group 3 with five representative secondary structures of each subgroup. Each of the subgroups of three groups (A1-A4, B1-B8, C1-C5) chooses a sequence as the representative to predict the secondary structure. Differences (mainly in bulges) are indicated with arrows.

Large-scale evaluation of the ITS1 rRNA secondary structure models exhibited that the majority of each subgroup within three groups shared the similar secondary structures (Fig 6). In specific, for Group 1 two subgroups (Fig 6A, A1 JX162769, and A2 FJ463918) exhibited the similar secondary structures with the representative model, while these two subgroups were all originated from India. And the other two subgroups (Fig 6A, A3 FJ216424, and A4 JQ520186) exhibited with the similar secondary structure with Group 2 model, while these two subgroups were all originated from China. Regarding Group 2, six subgroups shared the similar secondary structures with the slightly differences in bulges in helix 1 compared to model structure and they all originated from India (Fig 6B). Each of the other two subgroups (Fig 6B, B3 HM053465, and B4 GU726922) exhibited unique structures and originated from India. In addition, for Group 3, all the subgroups shared the similar structure with model structures (Fig 6C) except one subgroup exhibited a slightly different structure (Fig 6, C1 HQ222604). Three groups of ITS1 sequences exhibited obvious differences in helices of their secondary structure and the large-scale evaluations exhibited that for the majority of each subgroup, the ITS1 secondary structure could reflect originations of three groups of *Ganoderma lucidum*.

**Fig 7:**
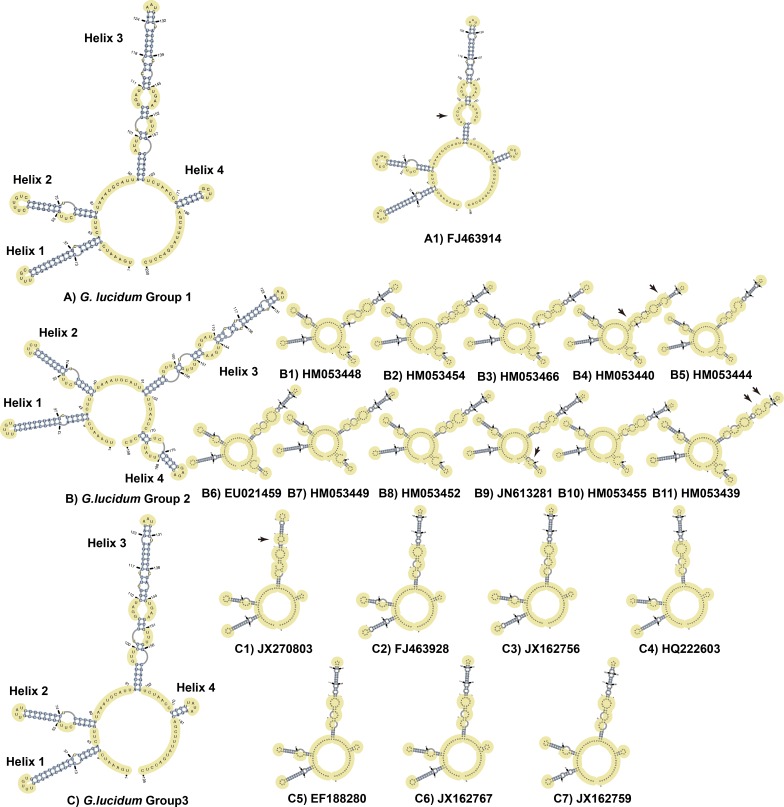
Large-scale evaluation of the ITS2 rRNA secondary structure models. A) *Ganoderma lucidum* Group 1 with one representative secondary structures of each subgroup. B) *Ganoderma lucidum* Group 2 with eleven representative secondary structure of each subgroup. C) *Ganoderma lucidum* Group 3 with seven representative secondary structures of each subgroup. Each of the subgroups of three groups (A1, B1-B11, C1-C7) chooses a sequence as the representative to predict the secondary structure. Differences (mainly in bulges) are indicated with arrows.

ITS2 sequences of three groups of *Ganoderma lucidum* were folded in the ITS2 database following the energy minimization method. Three representative structures are presented in Fig 5. For Group 1, 28 ITS2 sequences shared the same secondary structures (Fig 7A) except two sequences (FJ463914.1 and FJ463918.1) located in a sister group. Also for Group 2, 13 ITS2 sequences shared the same secondary structures (Fig 7B) which located in the main branch of Group 2 started from GQ249885.1 to HQ589218.1 and GQ249886.1 to HM053464.1. In addition, for Group 3, 71 ITS2 sequences shared the same secondary structures (Fig 7C) which located in the main branch of Group 3 started from JX162757.1 to GU213483.1, JQ520176.1 to JN222425.1, and JX162762.1 to GU213479.1. All these sequences in each group shared the same secondary structures. Therefore, a sequence from these sharing part of each group was selected as the representative to compare secondary structures and analyzed the nucleotide variance based on multi-alignments. The sequences used as representative secondary structure analysis for Group 1, Group 2, and Group3 were EU498090.1, EU021461.1, and FJ379263.1, respectively.

For ITS2 secondary structures, all three structures consisted of a central core formed by four helices. The central core of Group 1 consisted of three short helices (nt 8 to 43, 47 to 81 near the 5’ terminus and 171 to 186 near the 3’ terminus) and one long helix (nt 92 to 163) near the middle of the whole secondary structure. The central core of Group 2 consisted of three short helices (nt 8 to 43, 47 to 81 near the 5’ terminus and 170 to 195 near the 3’ terminus) and one long helix (nt 91 to 162) near the middle of the whole secondary structure. The central core of Group 3 shared the similar structures with Group 1, consisted of three short helices (nt 8 to 43, 47 to 80 near the 5’ terminus and 170 to 181 near the 3’ terminus) and one long helix (nt 91 to 162) near the middle of the whole secondary structure. The major patterns of nucleotide variances were C to U and G to A and *vice versa*.

Specifically, helix 1 of three *Ganoderma lucidum* groups was approximately 36 nt long and consisted of a long hairpin structure with one bulge and a small loop. The primary sequence of helix 1 was conserved in length. Furthermore, the 2D structure was also conserved, with only one or two variable nucleotides appearing in bulges (indicated in Fig 5) among the three groups of *Ganoderma lucidum*. Notably, hemi compensatory base change (hemi-CBC) site appeared in helix 1 of Group 1 (two hemi-CBC) and Group 3 (one hemi-CBC) and also one indel appeared in Group 1.

Helix 2 of three *Ganoderma lucidum* groups was approximately 35 nt long (Group 1) with only one nucleotide variance in length (Group 2 and Group 3) and consisted of a long hairpin structure with one bulge and a small loop. Furthermore, the 2D structure was conserved with only one or two variable nucleotides appearing in bulges (indicated in Fig 5) among the three groups of *Ganoderma lucidum*.

Helix 3 was the longest among all of the helices. This helix of three groups consisted of a long hairpin structure with six bulges and a small loop. The primary sequences of helix 2 in the three groups were conserved in length with 72nt. Variations of the 2D structure of helix 2 were observed with only one or two nucleotide polymorphisms among the three groups. Helix 2 contained one compensatory base change (CBC) in Group 1 and Group 2.

The primary sequences of helix 4 in the three groups were 16, 26 and 12 nt in length, respectively, including a short base-pair region, a bulge (Group 2), and a 4-nt loop. A non-CBC appeared in the base-pair region of Group 1. Helix 4 of Groups 1, 3, and 2 varied in sequence length from 11 (Group 3) to 26 nt (Groups 2). Bulge variances were also discovered (1 in Group 2). Notably, indels mainly appeared in helix 4 varied from 3 (Group 2) to 4 (Group 1) of three groups.

Large-scale evaluation of the ITS2 rRNA secondary structure models exhibited that compared to Group 2, Group 1 and Group 3 share the similar secondary structures. And the majority of each subgroup of these three groups shared the similar secondary structures (Fig 7). Group 1 and Group 3 shared the similar secondary structures. The only difference was the length of Helix 4. Regarding the similarity between Group 1 and Group 3, it was not recommended to utilize ITS2 secondary structure to reflect their origination.

### Strain antagonism analysis

**Fig 8:**
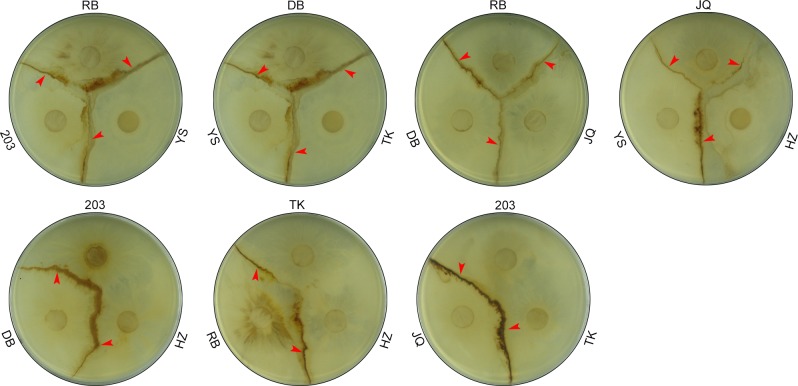
Antagonism tests of seven *Ganoderma lucidum* strains. The seven *Ganoderma lucidum* strains are indicated with different abbreviations (RB, YS, 203, DB, TK, JQ, and HZ). Antagonistic streaks are indicated with red arrows.

**Fig 9:**
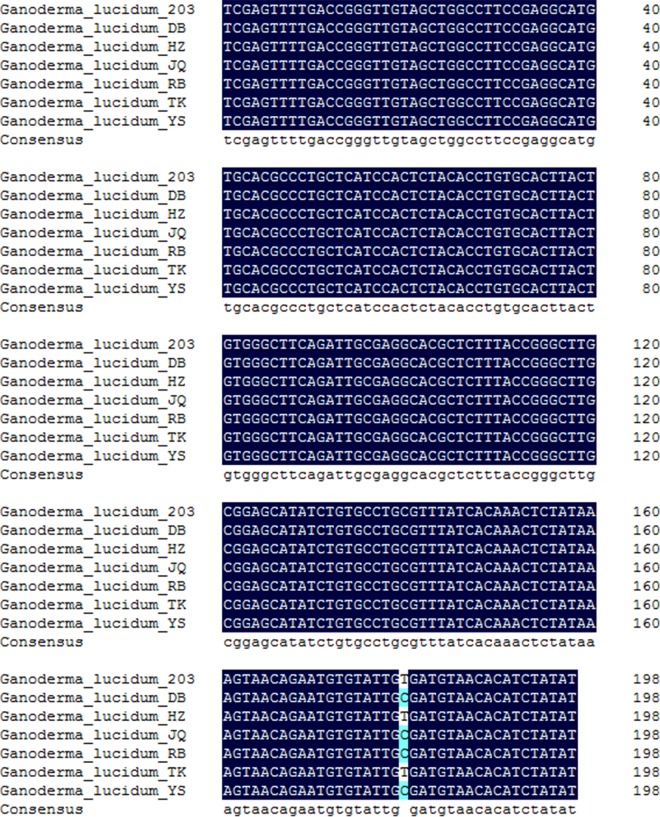
Multiple-alignment of *Ganoderma lucidum* ITS1 sequences. A single-nucleotide polymorphism is located at position 180. Cytosines are shaded with light blue, and thymines are shaded with white.

The seven *Ganoderma lucidum* spawn strains were differentiated in Group 3 with two subgroups according to ITS1 region sequences, while ITS2 region could only differentiate them in mainly Group 3 without subgroups. In order to test the differences among these strains, seven spawn strains were inoculated in accelerating medium plates (Fig 8) and exhibited different growth antagonism. A *Ganoderma lucidum* 203 (KX589244) fusion with *Ganoderma lucidum* TK (KX589249) and HZ (KX589246) exhibited intraspecific species similarities. However, other *Ganoderma lucidum* strains, including DB (KX589245), JQ (KX589247), RB (KX589248), and YS (KX589250), all exhibited antagonistic effects, and the antagonism streaks clearly present in Fig 8. indicating their genetic discrepancy. ITS1 alignment (Fig 9) revealed that the antagonism occurred when the position 180 was a T. ITS1 alignment revealed a C at position 180 in the antagonistic groups of *Ganoderma lucidum*. The SNP at 180th site in ITS1 correlated with the antagonistic test, which could subsequently identify *Ganoderma lucidum* strains at intraspecies level.

## Discussion

In this study, phylogenetic analyses with ITS1 sequences revealed that *Ganoderma lucidum* could be separated into three groups. Group 1 mainly originated from Europe and North America. Group 2 originated from India, Taiwan, and the Philippines. Group 3 originated from mainland China, Japan, and Korea. Phylogenetic analyses with ITS2 sequences were evaluated thus *Ganoderma lucidum* could be separated into two groups. Notably, Group 1 that mainly originated from Europe and North America was grouped together with another Group 3 that originated from mainland China, Japan, and Korea.

Similar to our results, Saltarelli et al. [57] tested *Ganoderma lucidum* isolates from Italy and China. Phylogenetic results divided *Ganoderma lucidum* into six groups. Group I originated from China and was phylogenetically distant from Groups IV and III, which originated from Europe and tropical Asia, respectively. The same results were also reported by Cao et al. [6]. Besides, higher fungi shared the same ITS2 secondary structure model with four helices and a central core and nucleotide evolve most rapidly in helix IV [58]. An attempt to use ITS2 as the barcoding method to differentiate *Ganoderma sp*. was previously reported. In their results, ITS2 could differentiate *Ganoderma lucidum* originated from Europe and Asia with *Ganoderma sinense* secondary structure comparison and most of *Ganoderma* species could be successfully identified using ITS2 sequences [37]. These results exhibited that ITS2 could differentiate *Ganoderma sp*. in species level and partially differentiate *Ganoderma lucidum* in intra-species level which was consistent with our results. However, our data showed that ITS1 could effectively distinguish *Ganoderma lucidum* strains, which reinforced the hypothesis that *Ganoderma lucidum* is a complex of species in which monophyletic groups correlate fairly well with their geographic origins [20,57].

The traditional taxonomy of the *Ganoderma* species is mainly based on the morphological characteristics of their fruit bodies, of which the most important are the shape and size of the basidiospores and cuticle cells [22]. However, *Ganoderma* species are easily misidentified due to their phenotypic plasticity. Therefore, different geographic locations or environmental conditions may result in polymorphisms of the basidiomes [20,59]. Strains of *Ganoderma lucidum* from Group 1 mainly originated from European countries. Several morphological features separate European *Ganoderma lucidum* from eastern Asian species. For example, in mature basidiocarps, *Ganoderma lucidum* from eastern Asia bears 1 to 2 black melanoid bands in the context and a yellow pore surface, whereas *Ganoderma lucidum* in Europe lacks a melanoid band structure and has a white pore surface [6]. In addition, the basidiocarps of *Ganoderma lucidum* from Asia have a slenderer stature than *Ganoderma lucidum* from Europe [60]. Consistent with a previous report from Moncalvo analyzing large subunit nuclear ribosomal DNA (LSU nrDNA) regions [20], our data also indicated that the collections of *Ganoderma lucidum* from Asia and Europe belong to different groups based on the analysis of their ITS1 sequences. Further, Moncalvo [20] also indicated that *Ganoderma lucidum* was distributed in northern and southern Europe and likely extended to China. Cao [6] confirmed that the distribution of *Ganoderma lucidum* included northeastern China. This finding could explain some *Ganoderma lucidum* nested in Group 1 that originated from China.

We found that *Ganoderma lucidum* strains from tropical Asia were nested in Group 2, whereas strains from mainland China, Korea and Japan were separated and clustered in Group 3. Similarly, Wang [60] also confirmed that *Ganoderma lucidum* in Asia represented at least two distinct species: one from Taiwan, India and the Philippines and the other from mainland China and Japan. Phylogenetic analysis revealed that the *Ganoderma lucidum* species that originated from tropical and eastern Asia were distinct species. Therefore, Wang believed that *Ganoderma lucidum* was a name mistakenly applied to Asian collections and that *Ganoderma multipileum* would be a more suitable name for *Ganoderma lucidum* in tropical Asia.

*Ganoderma lucidum* species from East Asia were nested in Group 3, including mainland China, Japan, and Korea. Interestingly, commercial spawn strains from mainland China were differentiated by two subgroups in Group 3, which exhibit only one nucleotide variance.

Within *Ganoderma* species, ITS regions are homogenized by the processes of concerted evolution and unequal crossing over. However, a growing number of ITS polymorphisms within a single individual have also been widely reported in fungi. For the ITS polymorphisms, some only occurred in the ITS1 region, whereas others may be found in the ITS2 region [39]. To further analyze the ITS1 and ITS2 sequences from *Ganoderma lucidum*, we aligned all of the sequences together and compared them using Weblogo, which showed that ITS1 harbored more nucleotide variance compared to ITS2 sequences. And ITS1 contained polymorphism sites mainly located at the center of the ITS1 region among the three groups. A similar study also found three highly variable regions: one in the central region of ITS1 and the others near the 5’ end of the ITS2 region [20]. Ancient hybridizations, gene duplications or low concerted evolution might be reasons for ITS1 heterogeneity among *Ganoderma lucidum* strains [39]. And these results further demonstrated that ITS1 exhibited the better performance compared to ITS2 sequences.

Determining a sequence’s RNA secondary structure can improve the accuracy and robustness in phylogenetic tree reconstruction [61]. Three secondary structures of ITS1 were identified based on phylogenetically differentiated groups and were correlated with their geographic distributions. The 2D structure of Group 1 that originated from Europe included four helices and a central core, whereas the other two groups consisted of three helices. Based on ITS1 sequence alignment, nucleotide variances were mainly indicated within the second helix and on the joint site of the central core with Helix 3 (Group 1) and Helix 4 (Groups 2 and 3), which may cause a slippery structure and helix unfolding. Inherent mechanisms involving slippage events during DNA duplication may cause minor intragenomic ITS heterogeneity. Such mechanisms may also involve indels or transitions of the ITS sequences from *Ganoderma* strains, as few sequence mutations occur in the ITS types [39]. Also, two types secondary structures of ITS2 were identified based on phylogenetically differentiated groups in which Group 1 and Group 3 shared similar models. The only difference of secondary structures between Group 1 and Group 3 was the length of Helix 4. These results were consistent with the phylogenetic analysis of ITS2 since these two groups formed the main group. Thus, the secondary structure of ITS1 significantly differentiates the species of *Ganoderma lucidum*, which could be robust evidence to support the phylogenetic analysis.

Molecular phylogenetic analysis with ITS nucleotide sequencing is simple and fast, but the identification of monophyletic groups does not always signify the identification of biological species [62]. Regarding the concept of biology species, if two species are compatible, they are grouped as one biological species [63]. Hyphal fusions reportedly occurred regularly in mycelia belonging to the same isolate, whereas barrage reactions would occur between higher fungi with different genetic origins [64]. Also, a vegetative compatible analysis was widely used in fungi to clarify the taxonomic and phylogenic relationships such as *Wolfiporia cocos* [65] and *Pleurotus sp*. [63]. Therefore, some reports have suggested that a vegetative compatibility test should be considered to identify *Ganoderma* isolates [23,24,66]. In addition, SNP sites in ITS2 regions of medical plants were recently used as species discrimination such as Szechuan Pepper [67] and Angelicae Sinensis Radix (Danggui) [68], which indicated that SNP sites in ITS region may be related to biological species. In our study, in order to discriminate seven commercial spawn strains of *Ganoderma lucidum* in mainland China, antagonism tests were performed. Three out of 7 exhibited compatibility and clustered in a subgroup in Group 3, while the others were incompatible. Interestingly, further multiple ITS1 alignments showed that a SNP located at site 180 was identified in seven *Ganoderma lucidum* strains. Three compatible strains contained T, and incompatible strains contained C. Our results showed that three *Ganoderma lucidum* compatible strains (203, HZ and TK, GenBank nos: KX589244, KX589246, KX589249, respectively) could be identical isolates, while the other four *Ganoderma lucidum* strains were not. Although incompatible strains were separated in Group 3 and shared identical ITS1 sequences, they are distinct *Ganoderma lucidum* isolates, and it is possible that they are not clonally derived, as proposed by Molina [69]. Our results showed that a SNP site in ITS1 correlated with the antagonistic test, which could subsequently identify *Ganoderma lucidum* strains.

In conclusion, our results showed that compared to ITS2, ITS1 phylogenetic and secondary structure analysis could differentiate *Ganoderma lucidum* into three geography distant groups. Compatibility testing and sequence alignments of commercial spawn *Ganoderma lucidum* strains of mainland China could distinguish identical biological species correlated with a SNP site in the ITS1 region. Our results propose a novel method for *Ganoderma lucidum* delineation, which will be implemented to improve species quality control in the *Ganoderma* industry.

## Supporting Information

S1 Table Taxa used in this study and their DNA sequences accession number in GenBank and publications. (DOCX)
