# Supplementary material for: Intraspecific Variation and Phylogenetic Relationships Are Revealed by ITS1 Secondary Structure Analysis and Single-Nucleotide Polymorphism in Ganoderma lucidum
Source: PLoS One. 2017 Jan 5;12(1):e0169042. doi: 10.1371/journal.pone.0169042 (PMC5215925; doi:10.1371/journal.pone.0169042)
Supplement: S1 Table — (DOCX) [file pone.0169042.s001.docx]

**S1 Table. Taxa used in this study and their DNA sequences accession number in GenBank and publications.**

| Species | strain | Geographic origin | Accession No. | Reference |
| --- | --- | --- | --- | --- |
| *Ganoderma lucidum* | XLLZ | - | JX162769 | Direct submission |
| *Ganoderma lucidum* | KR7 | India | FJ463918 | Direct submission |
| *Ganoderma lucidum* | KR8 | India | FJ463914 | Direct submission |
| *Ganoderma lucidum* | GL-01 | China | FJ216424 | [1] |
| *Ganoderma lucidum* | GL 166 | China | DQ425010 | [2] |
| *Ganoderma lucidum* | IUM-4242 | China | JQ520186 | [3] |
| *Ganoderma lucidum* | Cui 9207 | China | KJ143910 | [4] |
| *Ganoderma lucidum* | SC13091609 | - | KT318594 | Direct submission |
| *Ganoderma lucidum* | SC14090205 | - | KT318593 | Direct submission |
| *Ganoderma lucidum* | GI7099 | Italy | AM269773 | [5] |
| *Ganoderma lucidum* | GICN04 | Italy | AM906058 | [5] |
| *Ganoderma lucidum* | HMAS86597 | U.K. | AY884176 | [5] |
| *Ganoderma lucidum* | GLU5108 | Italy | EU498090 | [6] |
| *Ganoderma lucidum* | GL-04 | China | JN222422 | [7] |
| *Ganoderma lucidum* | Gl-4 | Armenia | JN588572 | [6] |
| *Ganoderma lucidum* | Gl-5 | Armenia | JN588573 | [6] |
| *Ganoderma lucidum* | GL-1-3 | France | JN588574 | [8] |
| *Ganoderma lucidum* | Glu1 | Italy | JN588575 | [8] |
| *Ganoderma lucidum* | Glu14 | Italy | JN588576 | Direct submission |
| *Ganoderma lucidum* | Glu15 | Italy | JN588577 | Direct submission |
| *Ganoderma lucidum* | Gl-1-2 | France | JN588578 | Direct submission |
| *Ganoderma lucidum* | ATCC 46755 | Canada | JQ520185 | [3] |
| *Ganoderma lucidum* | GRE3 | Poland | JQ627587 | [9] |
| *Ganoderma lucidum* | Dai2272 | Sweden | JQ781851 | [10] |
| *Ganoderma lucidum* | Glu2 | Italy | KJ509596 | [6] |
| *Ganoderma lucidum* | GL11 | China | DQ424972 | [2] |
| *Ganoderma lucidum* | AP2 | India | FJ463903 | Direct submission |
| *Ganoderma lucidum* | KL22 | India | FJ463911 | Direct submission |
| *Ganoderma lucidum* | KR13 | India | FJ463916 | Direct submission |
| *Ganoderma lucidum* | TN1 | India | FJ463922 | Direct submission |
| *Ganoderma lucidum* | TN27 | India | FJ463927 | Direct submission |
| *Ganoderma lucidum* | TN6 | India | FJ463930 | Direct submission |
| *Ganoderma lucidum* | 10-89 | Russia | GU207320 | Direct submission |
| *Ganoderma lucidum* | Al-1 | Russia | HM130563 | Direct submission |
| *Ganoderma lucidum* | Al-2 | Russia | HM130564 | Direct submission |
| *Ganoderma lucidum* | Al-3 | Russia | HM130565 | Direct submission |
| *Ganoderma lucidum* | Al-4 | Russia | HM130566 | Direct submission |
| *Ganoderma lucidum* | PF277 | Italy | JN176895 | [11] |
| *Ganoderma lucidum* | PF278 | Italy | JN176897 | [11] |
| *Ganoderma lucidum* | PF276 | Italy | JN176899 | [11] |
| *Ganoderma lucidum* | GL81 | Slovenia | KC311369 | Direct submission |
| S1 Table Continued |  |  |  |  |
| *Ganoderma lucidum* | JV 8510/21 | - | KF605614 | Direct submission |
| *Ganoderma lucidum* | JV 1208/9CH | - | KF605615 | Direct submission |
| *Ganoderma lucidum* | JV1208/1 | - | KF605616 | Direct submission |
| *Ganoderma lucidum* | BR 4195 | France | KJ143909 | [4] |
| *Ganoderma lucidum* | K 175217 | U.K. | KJ143911 | [4] |
| *Ganoderma lucidum* | MT 2610 | Czech Republics | KJ143912 | [4] |
| *Ganoderma lucidum* | Gl-B | - | KJ857252 | Direct submission |
| *Ganoderma lucidum* | Gl-7-2 | Armenia | KP941445 | Direct submission |
| *Ganoderma lucidum* | Glu 16 | Italy | KP941446 | Direct submission |
| *Ganoderma lucidum* | Glu 2 | Italy | KP941448 | Direct submission |
| *Ganoderma lucidum* | CBS 270.81 | France | Z37049 | [12] |
| *Ganoderma lucidum* | ATCC 46755 | USA | Z37052 | [12] |
| *Ganoderma lucidum* | RYV 33217 | Norway | Z37096 | [12] |
| *Ganoderma lucidum* | Gl-6 | Armenia | KP941444 | Direct submission |
| *Ganoderma lucidum* | Dai11593 | Finland | JQ781852 | [10] |
| *Ganoderma lucidum* | GL-34 | India | HM053457 | [13] |
| *Ganoderma lucidum* | GL-35 | India | HM053458 | [13] |
| *Ganoderma lucidum* | GL-36 | India | HM053459 | [13] |
| *Ganoderma lucidum* | GL-37 | India | HM053460 | [13] |
| *Ganoderma lucidum* | GL-38 | India | HM053461 | [13] |
| *Ganoderma lucidum* | GL-46 | India | HM053465 | [13] |
| *Ganoderma lucidum* | GL-45 | India | HM053464 | [13] |
| *Ganoderma lucidum* | GL-30 | India | HM053446 | [13] |
| *Ganoderma lucidum* | GL-32 | India | HM053448 | [13] |
| *Ganoderma lucidum* | GL-31 | India | HM053447 | [13] |
| *Ganoderma lucidum* | GL-50 | India | HM053454 | [13] |
| *Ganoderma lucidum* | GL-49 | India | HM053453 | [13] |
| *Ganoderma lucidum* | GL-42 | India | HM053452 | [13] |
| *Ganoderma lucidum* | GL-41 | India | HM053451 | [13] |
| *Ganoderma lucidum* | GL-13 | India | GU726927 | [13] |
| *Ganoderma lucidum* | GL-12 | India | GU726926 | [13] |
| *Ganoderma lucidum* | GL-51 | India | HM053455 | [13] |
| *Ganoderma lucidum* | GL-53 | India | JN099386 | [13] |
| *Ganoderma lucidum* | GL-52 | India | HM053456 | [13] |
| *Ganoderma lucidum* | BCRC 36123 | India | EU021459 | [14] |
| *Ganoderma lucidum* | BCRC 37043 | Taiwan | EU021460 | [14] |
| *Ganoderma lucidum* | OE-235 | China | AY636058 | [15] |
| *Ganoderma lucidum* | OE-233 | China | AY636068 | [15] |
| *Ganoderma lucidum* | ATCC 32472 | China | DQ425000 | [16] |
| *Ganoderma lucidum* | CWN01740 | Taiwan | EU021461 | [14] |
| *Ganoderma lucidum* | BCRC 37033 | Taiwan | EU021462 | [14] |
| *Ganoderma lucidum* | AP-6 | India | FJ463908 | Direct submission |
| *Ganoderma lucidum* | KR-3 | India | FJ463917 | Direct submission |
| S1 Table Continued |  |  |  |  |
| *Ganoderma lucidum* | TN-10 | India | FJ463920 | Direct submission |
| *Ganoderma lucidum* | TN-19 | India | FJ463925 | Direct submission |
| *Ganoderma lucidum* | CAW-23 | India | GQ249886 | [17] |
| *Ganoderma lucidum* | GL-1 | India | GU726919 | [13] |
| *Ganoderma lucidum* | GL-2 | India | GU726920 | [13] |
| *Ganoderma lucidum* | GL-3 | India | GU726921 | [13] |
| *Ganoderma lucidum* | GL-14 | India | HM053436 | [13] |
| *Ganoderma lucidum* | GL-15 | India | HM053437 | [13] |
| *Ganoderma lucidum* | GL-17 | India | HM053439 | [13] |
| *Ganoderma lucidum* | GL-16 | India | HM053438 | [13] |
| *Ganoderma lucidum* | GL-6 | India | GU726922 | [13] |
| *Ganoderma lucidum* | GL-7 | India | GU726923 | [13] |
| *Ganoderma lucidum* | GL-9 | India | GU726925 | [13] |
| *Ganoderma lucidum* | GL-8 | India | GU726924 | [13] |
| *Ganoderma lucidum* | GL-18 | India | GU726928 | [13] |
| *Ganoderma lucidum* | GL-19 | India | GU726929 | [13] |
| *Ganoderma lucidum* | GL-20 | India | GU726930 | [13] |
| *Ganoderma lucidum* | GL-21 | India | GU726931 | [13] |
| *Ganoderma lucidum* | GL-22 | India | GU726932 | [13] |
| *Ganoderma lucidum* | GL-23 | India | GU726933 | [13] |
| *Ganoderma lucidum* | GL-24 | India | HM053440 | [13] |
| *Ganoderma lucidum* | GL-25 | India | HM053441 | [13] |
| *Ganoderma lucidum* | GL-26 | India | HM053442 | [13] |
| *Ganoderma lucidum* | GL-27 | India | HM053443 | [13] |
| *Ganoderma lucidum* | GL-39 | India | HM053449 | [13] |
| *Ganoderma lucidum* | GL-40 | India | HM053450 | [13] |
| *Ganoderma lucidum* | GL-43 | India | HM053462 | [13] |
| *Ganoderma lucidum* | GL-44 | India | HM053463 | [13] |
| *Ganoderma lucidum* | GL-47 | India | HM053466 | [13] |
| *Ganoderma lucidum* | GL-48 | India | HM053467 | [13] |
| *Ganoderma lucidum* | AVK 1 | - | HM130706 | Direct submission |
| *Ganoderma lucidum* | 7 | - | KF998093 | Direct submission |
| *Ganoderma lucidum* | 8 | - | KF998094 | Direct submission |
| *Ganoderma lucidum* | 9 | - | KF998095 | Direct submission |
| *Ganoderma lucidum* | RSH RZ | Taiwan | X78743 | [12] |
| *Ganoderma lucidum* | ATCC 32471 | India | X78744 | [12] |
| *Ganoderma lucidum* | JMM P93-1 | Philippines | X78745 | [12] |
| *Ganoderma lucidum* | RSH G001 | Taiwan | X87345 | [18] |
| *Ganoderma lucidum* | RSH 0630 | Taiwan | X87346 | [18] |
| *Ganoderma lucidum* | RSH 0708 | Taiwan | X87347 | [18] |
| *Ganoderma lucidum* | RSH 0926 | Taiwan | X87348 | [18] |
| *Ganoderma lucidum* | RSH 0709 | Taiwan | X87349 | [18] |
| *Ganoderma lucidum* | RSH 0922 | Taiwan | X87350 | [18] |
| S1 Table Continued |  |  |  |  |
| *Ganoderma lucidum* | RSH 0626 | Taiwan | Z37048 | [18] |
| *Ganoderma lucidum* | ATCC 3472 | India | X87351 | [18] |
| *Ganoderma lucidum* | GIOE51 | - | HQ222604 | Direct submission |
| *Ganoderma lucidum* | Glstrain2 | - | HQ222603 | Direct submission |
| *Ganoderma lucidum* | G7 | China | JX162763 | [19] |
| *Ganoderma lucidum* | AP4 | India | FJ463907 | Direct submission |
| *Ganoderma lucidum* | KL1 | India | FJ463910 | Direct submission |
| *Ganoderma lucidum* | KL5 | India | FJ463912 | Direct submission |
| *Ganoderma lucidum* | TN32 | India | FJ463929 | Direct submission |
| *Ganoderma lucidum* | KL6 | India | FJ463919 | Direct submission |
| *Ganoderma lucidum* | AP12 | India | FJ463904 | Direct submission |
| *Ganoderma lucidum* | AP9 | India | FJ463909 | Direct submission |
| *Ganoderma lucidum* | TN2 | India | FJ463931 | Direct submission |
| *Ganoderma lucidum* | TN9 | India | FJ463921 | Direct submission |
| *Ganoderma lucidum* | WD565 | Japan | EU021455 | [14] |
| *Ganoderma lucidum* | WD-2308 | Japan | EU021456 | [14] |
| *Ganoderma lucidum* | 0537-8851472 | China | EU498091 | Direct submission |
| *Ganoderma lucidum* | GL-7 | China | FJ379262 | [20] |
| *Ganoderma lucidum* | GL99 | China | DQ424991 | [2] |
| *Ganoderma lucidum* | HG | China | FJ379265 | [21] |
| *Ganoderma lucidum* | SMCC170.01.27 | Korea | FJ501559 | [22] |
| *Ganoderma lucidum* | XZ-G-A1 | - | HQ235630 | Direct submission |
| *Ganoderma lucidum* | XZ-G-A2 | - | HQ235631 | Direct submission |
| *Ganoderma lucidum* | Yeongji-2 | Korea | JQ520169 | [8] |
| *Ganoderma lucidum* | IUM 0757 | Korea | JQ520175 | [8] |
| *Ganoderma lucidum* | IUM 0938 | Korea | JQ520176 | [8] |
| *Ganoderma lucidum* | IUM 4100 | Korea | JQ520179 | [8] |
| *Ganoderma lucidum* | ASI-7117 | Korea | JQ520180 | [3] |
| *Ganoderma lucidum* | GL-36 | China | DQ424984 | [2] |
| *Ganoderma lucidum* | GL-177 | China | DQ425015 | [2] |
| *Ganoderma lucidum* | GL-14 | China | GU213480 | [21] |
| *Ganoderma lucidum* | TK | China | KX589249 | This work |
| *Ganoderma lucidum* | HZ | Korea | KX589246 | This work |
| *Ganoderma lucidum* | 203 | China | KX589244 | This work |
| *Ganoderma lucidum* | SMCC170.01.28 | China | FJ501557 | [22] |
| *Ganoderma lucidum* | CSAAS0801 | China | FJ940919 | [23] |
| *Ganoderma lucidum* | GL-3B | China | GU213477 | [21] |
| *Ganoderma lucidum* | GL-23 | China | GU213484 | [21] |
| *Ganoderma lucidum* | GL-YW | China | GU213485 | [21] |
| *Ganoderma lucidum* | Gt-1 | China | GU213487 | [21] |
| *Ganoderma lucidum* | XZ-G-B | - | HQ235632 | Direct submission |
| *Ganoderma lucidum* | FCL188 | Japan | JN008869 | [9] |
| *Ganoderma lucidum* | FCL194 | Japan | JN008870 | [9] |
| S1 Table Continued |  |  |  |  |
| *Ganoderma lucidum* | FCL195 | Japan | JN008871 | [9] |
| *Ganoderma lucidum* | FCL197 | Japan | JN008872 | [9] |
| *Ganoderma lucidum* | FCL265 | Canada | JN222405 | [9] |
| *Ganoderma lucidum* | GLO1 | Japan | JN222421 | [9] |
| *Ganoderma lucidum* | FCL192 | Japan | JN222423 | [9] |
| *Ganoderma lucidum* | FCL196 | Japan | JN222424 | [9] |
| *Ganoderma lucidum* | GLO3 | Poland | JN222426 | [9] |
| *Ganoderma lucidum* | GLO2 | Poland | JN222425 | [9] |
| *Ganoderma lucidum* | GL-5 | China | JX162759 | [19] |
| *Ganoderma lucidum* | ASI-7004 | Korea | JQ520167 | [8] |
| *Ganoderma lucidum* | ASI-7013 | Korea | JQ520168 | [8] |
| *Ganoderma lucidum* | ASI-7074 | Korea | JQ520170 | [8] |
| *Ganoderma lucidum* | ASI-7091 | Korea | JQ520171 | [8] |
| *Ganoderma lucidum* | ASI-7094 | Korea | JQ520172 | [8] |
| *Ganoderma lucidum* | ASI 7135 | Korea | JQ520173 | [8] |
| *Ganoderma lucidum* | IUM-3986 | Korea | JQ520177 | [8] |
| *Ganoderma lucidum* | ASI-7037 | Korea | JQ520181 | [24] |
| *Ganoderma lucidum* | IUM-4303 | Bangladesh | JQ520182 | [24] |
| *Ganoderma lucidum* | IUM-4304 | Bangladesh | JQ520183 | [24] |
| *Ganoderma lucidum* | IUM-4310 | Bangladesh | JQ520184 | [24] |
| *Ganoderma lucidum* | KCTC 16802 | Thailand | JQ520188 | [24] |
| *Ganoderma lucidum* | ASI-7068 | - | JQ520189 | Direct submission |
| *Ganoderma lucidum* | FCL191 | Japan | JQ627589 | [9] |
| *Ganoderma lucidum* | FCL193 | Japan | JQ627590 | [9] |
| *Ganoderma lucidum* | 77002 | China | KF146177 | [25] |
| *Ganoderma lucidum* | WD565 | Japan | AB462322 | [26] |
| *Ganoderma lucidum* | GL-1 | China | DQ424969 | [2] |
| *Ganoderma lucidum* | GL-2 | China | DQ424970 | [2] |
| *Ganoderma lucidum* | GL-5 | China | DQ424971 | [2] |
| *Ganoderma lucidum* | GL-12 | China | DQ424973 | [2] |
| *Ganoderma lucidum* | GL-20 | China | DQ424974 | [2] |
| *Ganoderma lucidum* | GL-25 | China | DQ424979 | [2] |
| *Ganoderma lucidum* | GL-33 | China | DQ424981 | [2] |
| *Ganoderma lucidum* | GL-93 | China | DQ424983 | [2] |
| *Ganoderma lucidum* | GL-37 | China | DQ424985 | [2] |
| *Ganoderma lucidum* | GL-38 | China | DQ424986 | [2] |
| *Ganoderma lucidum* | GL-44 | China | DQ424987 | [2] |
| *Ganoderma lucidum* | GL-49 | China | DQ424988 | [2] |
| *Ganoderma lucidum* | GL-59 | China | DQ424989 | [2] |
| *Ganoderma lucidum* | GL-101 | China | DQ424992 | [2] |
| *Ganoderma lucidum* | GL-107 | China | DQ424993 | [2] |
| *Ganoderma lucidum* | GL-109 | China | DQ424994 | [2] |
| *Ganoderma lucidum* | ATCC 32471 | India | DQ424997 | [14] |
| S1 Table Continued |  |  |  |  |
| *Ganoderma lucidum* | GL146 | China | DQ425007 | [2] |
| *Ganoderma lucidum* | GL158 | China | DQ425008 | [2] |
| *Ganoderma lucidum* | GL161 | China | DQ425012 | [2] |
| *Ganoderma lucidum* | GL-174 | China | DQ425013 | [2] |
| *Ganoderma lucidum* | wjgl27 | China | EF188279 | [22] |
| *Ganoderma lucidum* | wjgl26 | China | EF188278 | [22] |
| *Ganoderma lucidum* | wjgl28 | China | EF188280 | [22] |
| *Ganoderma lucidum* | NW407 | China | EU520247 | Direct submission |
| *Ganoderma lucidum* | NH010 | - | FJ687271 | Direct submission |
| *Ganoderma lucidum* | Gam-1 | China | GU213471 | [21] |
| *Ganoderma lucidum* | Gl-3 | China | GU213476 | [21] |
| *Ganoderma lucidum* | Gl-8 | China | GU213478 | [21] |
| *Ganoderma lucidum* | Gl-10 | China | GU213479 | [21] |
| *Ganoderma lucidum* | Gl-20 | China | GU213481 | [21] |
| *Ganoderma lucidum* | Gl-22 | China | GU213483 | [21] |
| *Ganoderma lucidum* | Cui9164 | China | JN048774 | [27] |
| *Ganoderma lucidum* | Han G | China | JX162764 | [19] |
| *Ganoderma lucidum* | GL9 | - | KC311368 | Direct submission |
| *Ganoderma lucidum* | GL86 | - | KC311370 | Direct submission |
| *Ganoderma lucidum* | GL95 | - | KC311371 | Direct submission |
| *Ganoderma lucidum* | AEM14 | - | KM269294 | Direct submission |
| *Ganoderma lucidum* | ACCC 5.65 | China | X87354 | [14] |
| *Ganoderma lucidum* | HMAS 60537 | China | Z37050 | [12] |
| *Ganoderma lucidum* | YS | China | KX589250 | This work |
| *Ganoderma lucidum* | DB | China | KX589245 | This work |
| *Ganoderma lucidum* | JQ | China | KX589247 | This work |
| *Ganoderma lucidum* | RB | Japan | KX589248 | This work |
| *Ganoderma lucidum* | Gl-30 | China | DQ424980 | [2] |
| *Ganoderma lucidum* | 263-209 | Japan | AB509616 | Direct submission |
| *Ganoderma lucidum* | NC-8349 | - | AY456341 | [28] |
| *Ganoderma lucidum* | wjgl19 | China | EF188277 | Direct submission |
| *Ganoderma lucidum* | NW408B | China | EU520235 | Direct submission |
| *Ganoderma lucidum* | Gl-16 | China | FJ379263 | [29] |
| *Ganoderma lucidum* | TN31 | India | FJ463928 | Direct submission |
| *Ganoderma lucidum* | CAW-1 | India | GQ249880 | [30] |
| *Ganoderma lucidum* | CAW-17 | India | GQ249884 | [30] |
| *Ganoderma lucidum* | CAW-18 | India | GQ249885 | [30] |
| *Ganoderma lucidum* | CIRM-BRFM 885 | France | GU731558 | [31] |
| *Ganoderma lucidum* | GL-28 | India | HM053444 | [13] |
| *Ganoderma lucidum* | GL-29 | India | HM053445 | [13] |
| *Ganoderma lucidum* | CAW-29 | India | HQ589218 | [30] |
| *Ganoderma lucidum* | RCK 2011 | - | JN613281 | Direct submission |
| *Ganoderma lucidum* | IUM 0047 | Korea | JQ520174 | [3] |
| S1 Table Continued |  |  |  |  |
| *Ganoderma lucidum* | IUM-4002 | Korea | JQ520178 | [3] |
| *Ganoderma lucidum* | GL-1 | - | JX162754 | Direct submission |
| *Ganoderma lucidum* | GL-2 | China | JX162755 | [19] |
| *Ganoderma lucidum* | GG | China | JX162756 | [19] |
| *Ganoderma lucidum* | GL-3 | China | JX162757 | [19] |
| *Ganoderma lucidum* | Tai-1 | China | JX162762 | [19] |
| *Ganoderma lucidum* | GL-A | - | JX162767 | Direct submission |
| *Ganoderma lucidum* | CP382 | - | JX270802 | [32] |
| *Ganoderma lucidum* | CP145 | - | JX270803 | [32] |
| *Ganoderma lucidum* | MDU-3 | - | KC807228 | Direct submission |
| *Ganoderma lucidum* | BRFM<FRA>:953 | France | KF963254 | [31] |
| *Trametes versicolor* | CTB 863 A | Germany | EF524049 | [33] |
| *Trametes versicolor* | KUC8714 | Korea | HM008935 | [34] |

**References**

1. Gao X, Yao Q, Yang R, Wang S, Mu L, Liu L. Molecular identification of one wild Ganoderma strain Gl-01 and its characteristics of selenium accumulation. China Brewing. 2009;3(204):47-49. doi: 10.3969/j.issn.0254-5071.2009.03.014

2. Su C, Tang C, Zhang J, Chen M, Pan Y. The phylogenetic relationship of cultivated isolates of Ganoderma in China inferred from nuclear ribosomal DNA ITS sequences. Acta Microbiologica Sinica. 2007;47(1):11-16. doi: 10.13343/j.cnki.wsxb.2007.01.004

3. Park YJ, Kwon OC, Son ES, Yoon DE, Han W, Nam JY, et al. Genetic diversity analysis of Ganoderma species and development of a specific marker for identification of medicinal mushroom Ganoderma lucidum. Afr J Microbiol Res. 2012;6(25):5417-5425. doi: 10.5897/Ajmr12.846

4. Zhou LW, Cao Y, Wu SH, Vlasak J, Li DW, Li MJ, et al. Global diversity of the Ganoderma lucidum complex (Ganodermataceae, Polyporales) inferred from morphology and multilocus phylogeny. Phytochemistry. 2015;114:7-15. doi: 10.1016/j.phytochem.2014.09.023

5. Guglielmo F, Gonthier P, Garbelotto M, Nicolotti G. A PCR-based method for the identification of important wood rotting fungal taxa within Ganoderma, Inonotus s.l. and Phellinus s.l. Fems Microbiology Letters. 2008;282(2):228-237. doi: 10.1111/j.1574-6968.2008.01132.x

6. Saltarelli R, Ceccaroli P, Iotti M, Zambonelli A, Buffalini M, Casadei L, et al. Biochemical characterisation and antioxidant activity of mycelium of Ganoderma lucidum from Central Italy. Food Chemistry. 2009;116(1):143-151. doi: 10.1016/j.foodchem.2009.02.023

7. Wiater A, Paduch R, Choma A, Pleszczyńska M, Siwulski M, Dominik J, et al. Biological study on carboxymethylated (1 → 3)-α-d-glucans from fruiting bodies of Ganoderma lucidum. Int J Biol Macromol. 2012;51(5):1014-1023. doi: 10.1016/j.ijbiomac.2012.08.017

8. Kwon O, Park Y, Kim H, Kong W, Cho J, Lee C. Taxonomic position and species identity of the cultivated Yeongji ' Ganoderma lucidum' in Korea. Mycobiology. 2016;44(1):1-6. doi: 10.5941/myco.2016.44.1.1

9. Pawlik A, Janusz G, Debska I, Siwulski M, Frac M, Rogalski J. Genetic and Metabolic Intraspecific Biodiversity of Ganoderma lucidum. Biomed Res Int. 2015. doi: Artn 72614910.1155/2015/726149

10. Cao Y, Wu SH, Dai YC. Species clarification of the prize medicinal Ganoderma mushroom "Lingzhi". Fungal Divers. 2012;56(1):49-62. doi: 10.1007/s13225-012-0178-5

11. De Simone D, Annesi T. Occurrence of Ganoderma adspersum on Pinus pinea. Phytopathol Mediterr. 2012;51(2):374-382. doi: 10.14601/Phytopathol_Mediterr-9813

12. Moncalvo JM, Wang HF, Hseu RS. Gene phylogeny of the Ganoderma lucidum complex based on ribosomal DNA sequences. Comparison with traditional taxonomic characters. Mycological Research. 1995;99:1489-1499. doi:10.1016/S0953-7562(09)80798-3

13. Mohanty PS, Harsh NSK, Pandey A. Molecular phylogeny of Ganoderma lucidum isolates collected from northern India. Forest Pathology. 2012;42(5):429-436. doi: 10.1111/j.1439-0329.2012.00778.x

14. Wang DM, Wu SH, Su CH, Peng JT, Shih YH, Chen L-C. Ganoderma multipileum, the correct name for 'G. lucidum' in tropical Asia. Bot Stud. 2009;50(4):451-458

15. Singh S, Yadhav M, Upadhyay R, Kamal S, Rai R, Tewari R. Molecular characterization of specialty mushroom germplasm of the National Mushroom Repository. Mushroom Research. 2003;12(2):67-78

16. Tang C, Su C, Zhang J, Yang Y, Tang Q, Jia W, et al. Assemblage of 38 Ganoderma Strains Based on Combined rDNA ITS and Partial β-Tubulin Gene Sequences. shi yong jun xue bao. 2012;19(3):37-41

17. Rajesh K, Dhanasekaran D, Panneerselvam A. Isolation and taxonomic characterization of medicinal mushroom Ganoderma spp. Acad J Microbiol Res. 2014;2(2):61-70. doi: 10.15413/ajmr.2014.0109

18. Hseu RS, Wang HH, Wang HF, Moncalvo JM. Differentiation and grouping of isolates of the Ganoderma lucidum complex by random amplified polymorphic DNA-PCR compared with grouping on the basis of internal transcribed spacer sequences. Applied and Environmental Microbiology. 1996;62(4):1354-1363

19. Zhang X, Xu X, Liu H. Establishment of molecular ID in 11 Ganoderma lucidum strains. Microbiology. 2013;40(2):249-255. doi: 10.13344/j.microbiol.china.2013.

02.003

20. Kinge TR, Mih AM. Ganoderma ryvardense sp nov associated with basal stem rot (BSR) disease of oil palm in Cameroon. Mycosphere. 2011;2(2):179-188

21. Huang L, Yang X, Zhang Z, Hu H, Zhang Y, Wu Q. Identification of Ganoderma Spawns Based on ITS Sequence Analysis. Edible Fungi of China. 2010;29(1):55-57. doi: :10.13629/j.cnki.53-1054.2010.01.00

22. Jia D, Wang B, Peng W, Huang Z, Tan W, Gan B, et al. Analysis of ITS Sequences in 4 Wild Ganoderma lucidum Strains. Southwest China Journal of Agricultural Sciences. 2012;25(4):1414-1416. doi: 10.16213/j.cnki.scjas.2012.04.057

23. Tang H, Su Y, Lei Z, Li Z, Li X. Identification of medicinal polypore using DNA barcoding. World Chinese Medicine. 2016;11(5):771-775. doi:10.3969/j.issn.1673-7202.2016.01.005

24. Park Y-J, Kwon OC, Son E-S, Yoon D-E, Han W, Yoo Y-B, et al. Taxonomy of Ganoderma lucidum from Korea Based on rDNA and Partial beta-Tubulin Gene Sequence Analysis. Mycobiology. 2012;40(1):71-75. doi: 10.5941/myco.2012.40.1.071

25. Fang ZM, Liu XM, Chen LY, Shen Y, Zhang XC, Fang W, et al. Identification of a laccase Glac15 from Ganoderma lucidum 77002 and its application in bioethanol production. Biotechnology for Biofuels. 2015;8. doi: ARTN 5410.1186/s13068-015-0235-x

26. Sotome K, Matozaki T, Aimi T, Boonlue S. Polyporus thailandensis, a new species of group Polyporellus in Polyporus (Polyporales, Agaricomycota) from Northeastern Thailand. Mycoscience. 2016;57(2):85-89. doi: 10.1016/j.myc.2015.07.006

27. Zhao CL, Cui BK, Steffen KT. Yuchengia. a new polypore genus segregated from Perenniporia (Polyporales) based on morphological and molecular evidence. Nord J Bot. 2013;31(3):331-338. doi: 10.1111/j.1756-1051.2012.00003.x.

28. Edwards IP, Cripliver JL, Gillespie A, Johnsen KH, Scholler M, Turco RF. Nitrogen availability alters macrofungal basidiomycete community structure in optimally fertilized loblolly pine forests. New phytologist. 2004;162:755-770. doi: 10.1111/j.1469-8137.2004.01074.x

29. Huang LH, Yang XB, Hu HP, Zhang JM, Wu QP. Application of rDNA partial sequences in studies of edible fungi evolution. Chinese Journal of Health Laboratory Technology. 2011;21(7):1607-1610

30. Singh SK, Doshi A, Pancholy A, Pathak R. Biodiversity in wood-decay macro-fungi associatedwith declining arid zone trees of India as revealedby nuclear rDNA analysis. Eur J Plant Pathol. 2013;136:373-382. doi: 0.1007/s10658-013-0172-0

31. Welti S, Moreau PA, Decock C, Danel C, Duhal N, Favel A, et al. Oxygenated lanostane-type triterpenes profiling in laccate *Ganoderma* chemotaxonomy. Mycol Progress. 2015;14:45. doi: 10.1007/s11557-015-1066-7

32. Guerrero-Torres JV, Mata G, Martinez-Carrera D, Garibay-Orijel C, Garibay-Orijel yR. Primers for (1,3)-β-glucan synthase gene amplification and partial characterization of the enzyme in *Ganoderma lucidum*. Rev Iberoam Micol. 2013;30(4):267-270. doi: 10.1016/j.riam.2012.12.006

33. Kinge TR, Mih AM. Ganoderma ryvardense sp nov associated with basal stem rot (BSR) disease of oil palm in Cameroon. Mycosphere. 2011;2: 179-188

34. Cao Y, Yuan HS. Ganoderma mutabile sp nov from southwestern China based on morphological and molecular data. Mycol Prog. 2013;12: 121-126. doi: 10.1007/s11557-012-0819-9
